# The Noma Fund: an interview with Albert Roger Milla

**DOI:** 10.2144/fsoa-2020-0043

**Published:** 2020-05-28

**Keywords:** noma disease, foundation, fund

## Abstract

Albert Roger Milla speaks to Joseph Martin, Managing Editor: Milla is a former international football player for the Cameroon national team. More recently he is the president of the Noma Fund, an association aiming to completely eradicate Noma disease created by Milla, Joseph-Antoine Bell and Georges-Barthélémy Nko'Ayissi.

Find out more about the disease, the association and the president behind the project in this interview.

## You are the president of the Noma Fund Association. What does that position entail on a day-to-day basis?

It is common to think that association presidents only have an administrative role. For me, this is far from being the case.

Indeed, as president of the Noma Fund Association, my daily life is particularly busy. Since the creation of the association in January 2019, I have overseen its legal declaration, the organization of general meetings and everything related to communication of the association.

Website creation and the development of various communication tools and donation platforms are aspects that have required my daily involvement. I also travel a lot in order to meet the various actors who will be involved in the project. Indeed, the project ‘Acting against Noma 2021–2030’ carried out by Noma Fund, provides the deployment of means to fight against the disease in various territories, particularly on the African continent. I am, therefore, going to meet local political stakeholders who are at the very heart of the project’s success, to solicit their commitment. Finally, I am putting my personal and professional network at the service of the Noma cause. I meet with many different personalities on a daily basis, in order to make them aware of the disease, in the hope that they will join in the fight against Noma. In short, my days are full!

## Please can you tell us more about Noma?

Noma disease is caused by facial gangrene, which affects over 140,000 children aged 2–6 years annually in the poorest regions of the world, as a result of unhealthy living conditions, where access to healthcare as well as good health and nutritional practices are lacking. Noma initially manifests as small intra-oral lesions. These lesions, if not treated, develop into facial edema, putrid breath, destruction of the gum mucosa, lymph node involvement, high fever and diarrhea. At this stage, the infected child can still be treated. Otherwise, Noma progresses rapidly to a gangrenous form – the infection reaches the muscle and bone tissues. At this stage, childhood death occurs in 90% of cases.

This disease, which affects over 140,000 children aged 2–6 years each year, is still prevalent among the poorest populations in South America, South-East Asia and Africa.

## Why did you decide to get involved?

I became aware of Noma disease in February 2014. That year, I met many children in Cameroon who were victims of the disease: all of them had a tragic story and were oscillating between fatalism and hope. Indeed, without the existence of associations and actors who are committed to the prevention of Noma, the world’s most disadvantaged children affected by the disease are likely to die. Some of the children I met were waiting for treatment, others were waiting for funds for reconstructive surgery in Europe or America. Unfortunately, most of these children have since died, mainly due to a lack of means and investment in the fight against the disease.

Today, I am personally involved in the Noma Fund Association, in the hope of putting an end to these silent deaths.

## What are the main objectives of the Noma Fund Association?

To eradicate Noma, there are multiple objectives:

First, it is important to raise awareness throughout the at-risk populations. Providing information regarding Noma disease, its manifestations and its disastrous consequences, should allow us to significantly reduce the spread of the disease in the territories concerned.

Second, is preventative measures. This involves carrying out fieldwork in close collaboration with local stakeholders in order to ensure effective coverage against the disease. This involves the free distribution of food supplements to correct malnutrition, oral hygiene kits and antibiotics. These are simple, effective and relatively inexpensive measures, which will result in a significant reduction in the number of children affected by Noma.

Finally, there is an urgent need to provide care for Noma patients in their territory. Currently, Noma patients require significant financial resources to be able to travel to the specialist treatment centers located in Europe and America. Therefore, as a first step, we plan to build a regional reference hospital for Africa. This hospital, which will be located in Cameroon, will be able to receive all Noma patients, with a view to not only treating them, but also to envisage their social reintegration (maxillofacial repair, rehabilitation, etc.). In the future, we hope that the success of this project could be repeated in other areas affected by Noma disease, for example, South-East Asia and South America.

## How are you going to reach these objectives?

These are indeed ambitious objectives, which require the involvement of everyone. Therefore, we need to effectively pool human, logistical and material resources and gather as many people as possible to prevent the devastating effects of Noma.

We have already called on many international political figures, whom we have urged to join in the fight against the disease. We also plan to solicit and sensitize the political leaders of the G20 and UN member countries, so that they support us politically and financially in our objectives. This will enable us to facilitate the implementation of the measures required to reach our project goals, as defined in ‘Acting against Noma 2021–2030’.

The political leaders of the first ten target countries of the project also have an eminently central role to play. We want them to be truly involved in the project, so that the actions we plan to take can function optimally and sustainably. This goes hand in hand with the commitment of the various local actors (healthcare workers, associations, local figures, etc.), who will have a vital role to play in the implementation and achievement of the awareness and prevention objectives defined above.

Finally, we wish to make use of our networks, both personal and professional, in order to eradicate Noma. We hope that many celebrities will join us in this fight against the disease, which will help us give this cause the visibility and weight necessary to finally eradicate Noma.

## How can the public help?

The public can be a central actor in the success of the project ‘Acting against Noma 2021–2030’. The support they can provide can be both financial – through our donation campaigns – logistical and technical. By joining the Noma Fund Association, everyone can participate at their own level in raising awareness of Noma disease and helping children who are victims of the disease.

By sharing their commitment on social networks, spreading petitions about Noma disease or simply by raising awareness among their peers, the general public becomes an actor in the fight against Noma. It will really help to reduce the number of children affected by the disease.

Involving the general public is vital for the success of our project!

If you are interested in finding out more about the Noma Fund Association, information regarding the disease and actions can be found on the website and Facebook page [1,2]. Additionally, if you would like to sign the petition to support the implementation of the project ‘Acting against Noma 2021–2030’ then please do so here [3].

## You have had an amazing career – what would you say has been your biggest achievement?

I have indeed had the chance to experience many successes on the field. I’m still particularly proud of our performance at the 1990 World Cup. It was a real achievement, as Cameroon became the first African country to reach the quarterfinals at that time. We really had that feeling of carrying the whole African continent on our shoulders. It was a very strong feeling!

I can think of two games that come to mind, both at the 1990 World Cup: the first in the group phase against Romania, where I scored two goals. The second, in round 16 against Colombia. We really wanted to qualify! At the end of stoppage time, we couldn't break the deadlock at 1-1. It was in extra time that I scored my second goal, qualifying Cameroon for the quarterfinals. It was an extraordinary joy!

## What inspired you to do your trademark celebration at the 1990 World Cup?

It was a truly spontaneous, instinctive celebration, neither thoughtful nor calculated. I was overwhelmed with such joy that I rushed to the corner post. It became my very personal way of celebrating my goals afterward.

## What skills have you taken from your years as a professional football player that are applicable to your current role as president of the Noma Fund Association?

Although they are very different areas of competence and fields of action, some aspects are indeed in agreement. In order to carry out my mission as president of the Noma Fund Association, I must take up new challenges every day, solve complex problems and bring together as many people as possible. My qualities of endurance, tenacity and leadership – both on and off the field – are major assets. I also think that my career as a footballer allowed me to travel and meet many people, which makes me what I call a ”citizen of the world”, which must have impacted my commitment and influenced me in one way or another.

## References

[1] The Noma Fund Association (2020). https://noma-fund.org/en/

[2] The Noma Fund Association Facebook (2020). www.facebook.com/AssociationNomaFund/

[3] Acting against Noma 2021–2030. The Noma Fund Association, (2020). https://noma-petition.org/en/appeal-to-heads-of-state/

